# Study on the Pyrolysis and Adsorption Behavior of Activated Carbon Derived from Waste Polyester Textiles with Different Metal Salts

**DOI:** 10.3390/ma15207112

**Published:** 2022-10-13

**Authors:** Lun Zhou, Meng-Qi Zhong, Teng Wang, Jing-Xin Liu, Meng Mei, Si Chen, Jin-Ping Li

**Affiliations:** 1 School of Environmental Engineering, Wuhan Textile University, Wuhan 430073, China; 2 Engineering Research Centre for Clean Production of Textile Dyeing and Printing, Ministry of Education, Wuhan Textile University, Wuhan 430073, China

**Keywords:** Cr(VI), adsorption, waste polyester textile, pyrolysis behavior, metal salts, activated carbon

## Abstract

In this study, the effects of the catalysis of heavy metals on the pyrolysis of waste polyester textiles (WPTs) and the adsorption behaviors of the pyrolysis products of WPTs for Cr(VI) were explored. TG−DTG analysis indicated that the metal ions catalyzed the pyrolysis process by reducing the temperature of the decomposition of WPTs. The surface morphology and pore structure of the carbons were analyzed using SEM and BET. The results demonstrated that Zn−AC possessed the largest specific surface area of 847.87 m^2^/g. The abundant acidic functional groups on the surface of the activated carbons were proved to be involved in the Cr(VI) adsorption process via FTIR analysis. Cr(VI) adsorption experiments indicated that the adsorption process was more favorable at low pH conditions, and the maximum adsorption capacities of Zn−AC, Fe−AC, and Cu−AC for Cr(VI) were 199.07, 136.25, and 84.47 mg/g, respectively. The FTIR and XPS analyses of the carbons after Cr(VI) adsorption, combined with the adsorption kinetics and isotherm simulations, demonstrated that the adsorption mechanism includes pore filling, an electrostatic effect, a reduction reaction, and complexation. This study showed that metal salts catalyze the pyrolysis processes of WPTs, and the activated carbons derived from waste polyester textiles are promising adsorbents for Cr(VI) removal.

## 1. Introduction

With the popularity of fast fashion, masses of textiles were produced rapidly, which makes the lifecycles of textiles constantly shortened, resulting in abundant waste textiles produced in recent years [1]. More than 14 million tons of polyester textiles are discarded in China as waste every year [2]. Waste polyester textiles contain heavy metals, mainly from the heavy metal composite dyes used in the printing and dyeing process and the reinforcing agents containing heavy metals to achieve antibacterial, waterproof, and other such effects [3]. More than 69% of waste polyester textiles are treated in landfills [4], which causes heavy metal pollution of soil and groundwater. Therefore, it is urgent to find a suitable treatment method [5].

Chromium is one of the most hazardous heavy metal contaminants, which exists in nature in both trivalent and hexavalent oxidation states as the predominant forms. Cr(VI) is much more hazardous than other forms due to its higher mobility. Compared with the mildly soluble Cr(III), Cr(VI) has extremely strong bioavailability [6,7], which has carcinogenicity and mutagenicity to organisms. Furthermore, it can damage the liver and lungs of organisms, and cause skin ulceration [8]. Coagulation, precipitation−/redox−assisted coagulation, ion exchange, membrane treatment, and adsorption are the conventional methods for the removal of Cr(VI) from polluted water [9]. However, adsorption has been widely used because of its cost−effectiveness, sustainability, versatility, and easy operation [10].

Activated carbon (AC) is a contaminant adsorbent that has been extensively used in the environmental protection field in recent years, which is often used to absorb various metal pollutants in polluted water with the advantages of being cost−effective, renewable, and environmentally friendly [11,12]. It is usually prepared through pyrolysis and the carbonization of materials with high carbon content [13] and possesses a well−developed pore structure and various functional groups after activation [14]. However, the commonly used carbon element materials with high content such as coke and asphalt are nonrenewable, which makes the production cost of activated carbon relatively high. Therefore, searching for inexpensive and environmentally benign AC precursor materials is an important research direction. Recently, some of these materials have been proven that can be used to prepare AC, such as nut shells [15], Sugarcane bagasse [16], coconut coir [17], Bael fruit shell [18], tobacco petiole [19], etc. Meanwhile, more and more high−performance activated carbons have been prepared from inexpensive materials and have potential applications in more fields, such as battery noble metal removal [14,20,21].

Due to their huge quantity and rich carbon, WPTs can be considered ideal materials for the preparation of AC. At present, some researchers prepared AC through pyrolysis from WPTs and utilized it to adsorb heavy metals [2]. However, for common heavy metal contaminants, especially Fe, Zn, and Cu [3], which are commonly used as pyrolysis catalysts [22], few studies have been performed on the effects of the WPT pyrolysis process and the properties of pyrolysis products.

In this study, the raw material for the preparation of AC was waste polyester textile (WPT). Three major metal ions were added to WPTs by using the impregnation method. The catalytic effects of these heavy metal ions on the WPT pyrolysis process, the properties of different ACs, and the differences between these heavy metal ions in catalytic effects were studied. Cr(VI) adsorption experiments were performed on the ACs activated by the three heavy metal ions. The adsorption mechanism and adsorption behaviors of the ACs were analyzed, and the different effects of heavy metal active agents on the Cr(VI) adsorption behaviors of the ACs were discussed.

## 2. Materials and Methods

### 2.1. Materials

The WPTs used in this research were recovered from a textile mill in Nantong, Jiangsu Province, China. The primary chemicals in this research were zinc chloride (ZnCl_2_), anhydrous ferric chloride (FeCl_3_), copper(II) nitrate trihydrate (Cu(NO_3_)_2_·3H_2_O), and hydrochloric acid (HCl), which were purchased from Sinopharm Chemical Reagent Co. (Shanghai, China). Analytical pure−grade chemical reagents were used for all experiments, and ultrapure water was used to prepare the solutions for the experiments.

### 2.2. Methods

The pyrolysis behaviors of the samples were determined using a thermogravimetric–differential thermogravimetric (TG−DTG) analyzer (TG209F1) from 30 to 800 °C at a rate of 10 °C/min under an N_2_ atmosphere (with a flow rate of 100 mL/min). The surface functional group variations before and after modification/adsorption were measured using a Fourier transform infrared (FTIR) spectrometer (Thermo Fischer, Nicolet iS5, Waltham, MA, USA, resolution of 4 cm^−1^, 32 scans, from 400 to 4000 cm^−1^) and X−ray photoelectron spectroscopy (XPS; Thermo Fischer, ESCALAB 250XI, Waltham, MA, USA, Al Kα radiation, energy resolution of 1.0 eV). The surface morphologies and pore structures of the samples were characterized via scanning electron microscopy–energy−dispersive spectrometry (SEM−EDS; Zeiss Sigma 300, Oberkochen, Germany). The specific surface areas and aperture distributions of the samples were measured using the Brunauer–Emmett–Teller (BET; ASAP 2460, USA) method on the basis of the measurements relating to N_2_ adsorption with an apparatus at 77 K; each sample was degassed at 473 K for 12 h, and the pore volume was determined at *p*/*p*_0_ > 0.99.

The pH shift method [23] was used to measure the pH−dependent point zero charges (pH_PZC_) of the ACs: Briefly, 0.01 mol/L NaCl solutions were adjusted to the initial pH value (ranged from 2 to 8 in step−size of 1) using 0.1 mol/L NaOH and 0.1 mol/L HCl; subsequently, 0.025 g samples (AC, Zn−AC, Fe−AC, and Cu−AC) were added to 30 mL adjusted NaCl solutions and shaken in a shaker for 24 h (250 rpm, 25 °C). The final pH was measured, to obtain the ΔpH (the difference between the initial pH and the final pH), and then plotted against with initial pH. The experimental data were fitted using cubic spline interpolation, and the point at ΔpH = 0 on the curve gave pHpzc [24].

### 2.3. Sample Preparation

The WPTs were sheared into 3 × 3 mm fragments and then impregnated into a 50 mL ZnCl_2_, FeCl_3_, and Cu(NO_3_)_2_ solution with a mass ratio of 1:2 (WPT: ZnCl_2_/FeCl_3_/Cu(NO_3_)_2_) at room temperature for 24 h. The mixtures were put into a drying oven at 60 °C, dried for 12 h, and named Zn−WPT, Fe−WPT, and Cu−WPT, respectively. The dried WPTs were packed inside a quartz boat and loaded in a tube furnace, and then N_2_ was bubbled for 30 min into the furnace to exclude the air and form the N_2_ atmosphere. The temperature program was set as a heating rate of 10 °C/min, a final temperature of 800 °C, and a holding time of 1 h. After the tube furnace cooled to room temperature, the mixtures were taken out and treated by soaking with a 100 mL HCl solution (10% *v/v*) for 10 min, and then washed with deionized water until the washed water reached neutrality. The pyrolyzed mixtures were ground into powder (100 meshes) and placed in sealed bags for subsequent use. The ACs obtained by different treatment reagents (ZnCl_2_, FeCl_3_, and Cu(NO_3_)_2_) were named Zn−AC, Fe−AC, Cu−AC, and AC.

### 2.4. Adsorption Experiments

#### 2.4.1. Effect of pH

The initial pH value of the Cr(VI) solution (200 mg/L) was adjusted to the range of 2−8 using NaOH (0.1 mol/L) and HCl (0.1 mom/L), and each 0.025 g AC was put into 30 mL Cr(VI) solutions with different pH values. The adsorption reaction was carried out in a shaking bed at 150 rpm under 25 °C conditions. A 0.45 μm filter membrane was used to filter out the solution after shaking for 24 h. The concentration of the filtered solution was measured with a UV–visible spectrophotometer (UV−5500PC) at 540 nm, and the total Cr in the filtrate was determined by using the atomic absorption flame method (NOVAA−800, Germany). The Cr(VI) adsorption capacity (q) and Cr(VI) removal rate (R) was calculated as follows (Equations (1) and (2)):

(1) $$q=\frac{\left(C_{0}-C_{e}\right)\times V}{m}$$

(2) $$R=\frac{\left(C_{0}-C_{e}\right)}{C_{0}}\times 100\%$$

where C_0_ (mg/L) and C_e_ (mg/L) are the initial and equilibrium Cr(VI) concentrations, V (ml) is the solution volume, and m (g) is the dry weight of the adsorbent samples.

After adsorption, the sample was separated using vacuum filtration and dried for 24 h in a drying oven.

#### 2.4.2. Adsorption Kinetics

Each 0.025 g AC was put into 30 mL of the Cr(VI) solution (200 mg/L, pH = 2). The adsorption reaction and filtration of the solution were carried out under the same conditions as in Section 2.4.1. The solutions at different adsorption times (5 min~24 h) were sampled through filtration. Pseudo−first−order (PSO), pseudo−second−order (PFO), Elovich, and intra−particle diffusion models were used to fit the experimental data, which were expressed as follows:

PFO model:

(3) $$\ln\left(q_{e}-q_{t}\right)={\mathrm{lnq}}_{e}-k_{1}t$$

PSO model:

(4) $$\frac{t}{q_{t}}=\frac{1}{k_{2}q_{e}^{2}}+\frac{t}{q_{e}}$$

Elovich model:

(5) $$q_{t}=\frac{1}{b}\ln ab+\frac{1}{b}\ln t$$

Intra−particle diffusion model:

(6) $$q_{t}=k_{3}\sqrt{t}+C$$

where q_e_ (mg/g) and q_t_ (mg/g) are the Cr(VI) adsorption capacities at equilibrium and time t; k_1_ (min^−1^), k_2_ (g/(mg⋅min)) and k_3_ ((mg·min^0.5^/g)) are the rate constants of the pseudo−first−order model, pseudo−second−order model, and intra−particle diffusion model, respectively; a (mg/g⋅min) is the rate constant of chemisorption; b (g/mg) is the constant of the surface coverage; C (mg/g) is a constant.

#### 2.4.3. Adsorption Isotherms

Each 0.025 g sample was put into 30 mL of Cr(VI) solutions (pH = 2) with different initial concentrations (100~800 mg/L). The adsorption conditions and sampling methods were the same as those in Section 2.4.1. The experimental data were fitted with Langmuir and Freundlich isotherm models, and the equations are as follows:

Langmuir model:

(7) $$q_{e}=\frac{q_{m}K_{L}C_{e}}{1+K_{L}C_{e}}$$

Freundlich model:

(8) $$q_{e}=K_{F}C_{e}^{\frac{1}{n}}$$

where C_e_ (mg/g) is the equilibrium concentration of the Cr(VI) solution, q_e_ (mg/g) is the adsorption capacity at equilibrium, q_m_ (mg/g) is the maximum adsorption capacity, K_L_ (L/mg) is the Langmuir affinity constant, and n and K_F_ ((mg/g) (mg/L)^−1/n^) are the Freundlich constants corresponding to the adsorption intensity and capability, respectively.

## 3. Results

### 3.1. Pyrolysis Behavior

**Figure 1:**
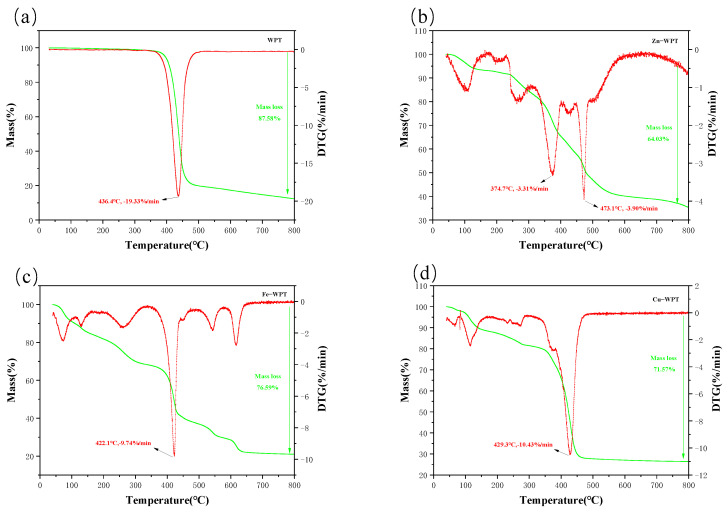
The TG−DTG curves of the four samples:−−−(**a**) WPT; (**b**) Zn−WPT; (**c**) Fe−WPT; (**d**) Cu−WPT.

Figure 1 shows the TG−DTG analysis results of the pyrolysis process of WPT, Zn−WPT, Fe−WPT, and Cu−WPT. According to Figure 1a, the mass loss processes of the WPT during pyrolysis could be regarded in three stages. The temperature range of the first stage was from 40 °C to 370 °C, where the mass loss rate was 2%, and this was attributed to the volatilization of small molecule substances and carbon dioxide [25,26]. The main mass loss stage was in the temperature range of 370~470 °C, where the mass loss rate was 76%. Due to the decomposition of WPTs to generate complex carbocyclic molecules [27], an obvious peak appeared in the DTG curve at 436 °C. The third stage was the heating stage of 500~800 °C, with no significant mass loss.

Figure 1b shows that the mass loss process of Zn−WPT mainly included three stages. The first stage (40~300 °C) was mainly the volatilization of the small molecular substances and carbon dioxide of Zn−WPT and the dehydration effect of ZnCl_2_ [28], with a mass loss rate of 15.2%. The second stage (300~600 °C) was mainly the stage of the catalytic decomposition of Zn−WPT. The release of small molecular species was responsible for the appearance of a peak at 375 °C in the DTG curve [26]. The temperature at which the peak appeared was lower than that of WPT, indicating that Zn^2+^ decreased the decomposition temperature. Subsequently, ZnCl_2_ was vaporized and decomposed, and WPT was decomposed to generate complex carbocyclic molecules, and a peak could be observed in the DTG curve at 473 °C, with a mass loss of 37.9%. In the third stage (600~800 °C), the residual solid in the second stage was further pyrolyzed and carbonized to form a stable structure and released volatiles, with a mass loss rate of 5%.

According to Figure 1c, the pyrolysis process of Fe−WPT included three stages. In the first stage (40~170 °C), corresponding to the decomposition of FeCl_3_ into FeOOH and HCl (g), HCl (g) could catalyze the pore formation process [27], and the mass loss in this stage was about 16.2%. In the second stage (170~500 °C), an obvious peak could be observed at about 260 °C in the DTG curve, which was due to the iron salts undergoing decomposition and reduction reactions to form iron oxides [29]. Then, the main components of Fe−WPT decomposed rapidly, and a peak appeared in the DTG curve at 422 °C, which was lower than the peak of WPT but higher than that of Zn−WPT, with the mass loss of about 31.6% in the whole stage. The mass loss in the third stage (500~800 °C) was about 13.5%, which was mainly caused by the consumption of carbon with Fe_2_O_3_ and Fe_3_O_4_ through the reduction reaction, and the reaction between Fe and amorphous carbon generated Fe_3_C [30]. The main reactions of Fe in different stages are as follows [27,28,29,30]:

First stage:

(9) $${\mathrm{FeCl}}_{3}+2H_{2}O\;\to \;\mathrm{FeOOH}\;+3\mathrm{HCl}\;\left(g\right)$$

Second stage:

(10) $$2\mathrm{FeOOH}\;\to {\;\mathrm{Fe}}_{2}O_{3}+{\;H}_{2}O\;\left(g\right)$$

(11) $${\mathrm{Fe}}_{2}O_{3}+\;C\left(\mathrm{CO}\right)\;\to \;2{\mathrm{Fe}}_{3}O_{4}+\;\mathrm{CO}\left({\mathrm{CO}}_{2}\right)$$

Third stage:

(12) $$2{\mathrm{Fe}}_{2}O_{3}+3C\;\to \;4\mathrm{Fe}\;+3{\mathrm{CO}}_{2}$$

(13) $${\mathrm{Fe}}_{3}O_{4}+2C\;\to \;3\mathrm{Fe}\;+2{\mathrm{CO}}_{2}$$

(14) $$\mathrm{Fe}\;+\;\mathrm{amorphous}\;\mathrm{carbon}\to {\mathrm{Fe}}_{3}C$$

As presented in Figure 1d, there were three stages in the pyrolysis process of Cu−WPT. The mass loss of about 12.3% in the first stage (40~300 °C) was mainly due to the volatilization of the small molecular substances of Cu−WPT and the decomposition of Cu(NO_3_)_2_. Then, Cu−WPT was quickly thermally decomposed and released numerous light volatile components in the second stage (300~480 °C), with a peak appearing in the DTG curve at around 429 °C, which was slightly lower than that of WPT, and the mass loss at this stage was 53.6%. In the third stage (480~800 °C), the residues slowly decomposed and carbonized, and the mass loss rate was 1.5%.

According to the DTG curves, the residual mass values at 800 °C for the four samples were 12.42%, 35.97%, 23.41%, and 28.43%, respectively. The residual mass values of the three impregnated samples were higher than that of the bare WPT, which may be due to the deposition of metal compounds on the surface of activated carbon during the pyrolysis process. The maximum mass loss temperatures of Zn−WPT, Fe−WPT, and Cu−WPT were 375 °C, 422 °C, and 429 °C, respectively, which were lower than 436 °C of the bare WPT. This showed that the addition of Zn^2+^, Fe^3+^, and Cu^2+^ could reduce the temperature requirements for the decomposition of WPTs, and Zn^2+^ had the most significant effect.

### 3.2. Characterization of AC

#### 3.2.1. SEM and BET

**Figure 2:**
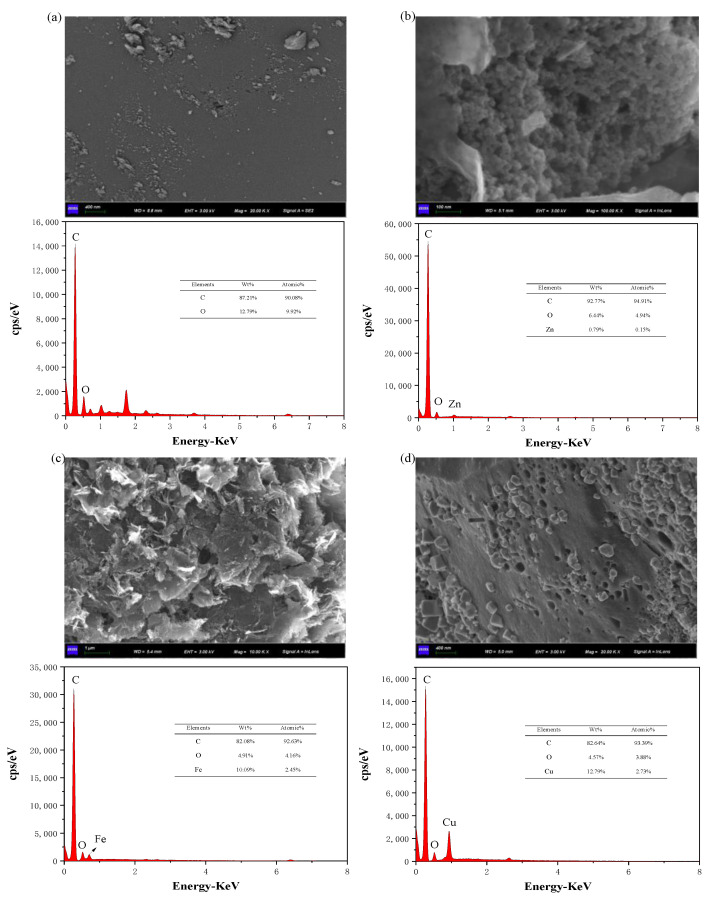
The SEM−EDS maps of the four samples: (**a**) AC; (**b**) Zn−AC; (**c**) Fe−AC; (**d**) Cu−AC.

The SEM micrographs of the samples are shown in Figure 2. According to Figure 2a, the surface of the AC was relatively smooth, and no obvious pore structure appeared. From Figure 2b–d, it can be inferred that the morphology of the ACs was obviously changed after activation, and the surface of Zn−AC, Fe−AC, and Cu−AC became rough and uneven, with an obvious pore structure. In addition, there were many particles on the surfaces of Cu−AC and Fe−AC, while only a few particles remained on the surface of Zn−AC. This may be due to the fact that the compounds related to Zn were easily dissolved in hydrochloric acid solutions, whereas those related to Cu and Fe were relatively more difficult to be dissolved. The dissolution of particles favored the development of pore structure on the surfaces of the ACs, and thus the surface of Zn−AC formed more pores. The EDS spectra in Figure 2 show the main components of the samples: The components of the AC were mainly C and O, and the wt% values were 87.21% and 12.79%, respectively; the components of Zn−AC were mainly C, O, and Zn, with wt% of 92.77%, 6.44%, and 0.79%, respectively; the components of Fe−AC were mainly C, O, and Fe, with wt% of 82.08%, 4.91%, and 10.09%, respectively; Cu−AC were mainly consisted of C, O, and Cu, with wt% of 82.64%, 4.57%, and 12.79%, respectively. The low content of Zn indicated that most of the ZnCl_2_ had evaporated or dissolved in a hydrochloric acid solution during pyrolysis. The higher content of Fe and Cu showed that part of Fe and Cu deposited on the surface of the AC during the catalytic pyrolysis reaction and was less dissolved in hydrochloric acid solution. This result was consistent with the SEM images.

From Table 1, the specific surface areas of AC, Zn−AC, Fe−AC, and Cu−AC were 548.89, 847.87, 229.66, and 98.33 m^2^/g, respectively. Due to the attachment of metal oxide on the surface and pores of AC, and because high−temperature nitrogen caused the loosening of carbon structure on the surface, resulting in some micropores being blocked [31], the specific surface area of the AC decreased after its activation by FeCl_3_ and Cu(NO_3_)_2_. In addition, this causes poorly developed spatial crosslinks in the samples while the metal salt solution reaches a certain concentration, which may hinder the reaction between the catalyst and the carbon structure, resulting in serious external loss and the slow growth of micropores on the surfaces of the ACs [30]. The microporous proportions of AC, Zn−AC, and Cu−AC were 94.03%, 80.20%, and 96.26%, respectively. These indicated that AC, Zn−AC, and Cu−AC mainly possessed microporous structures. The value of the S_ext_/S_BET_ of Fe−AC was 55.84%, which indicated that there may be more mesopores in Fe−AC. The total pore volumes (V_t_) of AC, Zn−AC, Fe−AC, and Cu−AC were 0.213, 0.452, 0.452, and 0.043 cm^3^/g, respectively, while the micropore volumes (V_mic_) were 0.197, 0.274, 0.043, and 0.039 cm^3^/g, respectively. The average pore diameters (D_P_) of the four materials were 1.55, 2.13, 7.87, and 1.74 nm, respectively, which were larger than the diameter of Cr(VI) (0.104 nm). Therefore, the pore structures of these four materials all satisfied the conditions for Cr(VI) adsorption.

**Figure 3:**
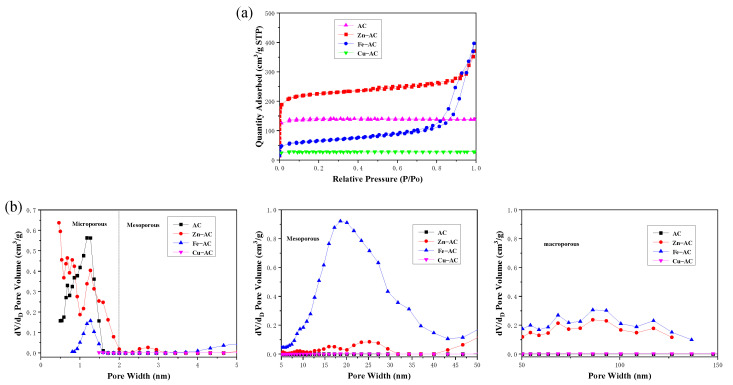
(**a**) The N_2_ adsorption/desorption isotherms and (**b**) pore size distribution for the samples.

The N_2_ adsorption/desorption isotherm and the corresponding pore size distributions of each sample are shown in Figure 3. According to Figure 3a, both the AC and Cu−AC displayed a type−I isotherm without hysteresis. Combined with the values of S_mic_/S_BET_, this indicated that the pore structure of the AC and Cu−AC were mainly microporous. Both Fe−AC and Zn−AC displayed a type−IV isotherm with an H_3_ hysteresis loop, indicating that the pore structures were mesoporous, and the pores were mainly slit−shaped [32]. Combined with the microporous proportions, the pore structure of Zn−AC may be micro−mesoporous. When *p*/*p_0_* < 0.4, Fe−AC showed weak N_2_ adsorption. This illustrated that Fe−AC had almost no micropores and was mainly associated with a mesoporous structure. The pore size distributions of all the samples are displayed in Figure 3b. The calculated porosity values of AC, Zn−AC, Fe−AC, and Cu−AC were 58.78%, 75.75%, 73.17%, and 21.96%, respectively, which were calculated using the porosity calculation equation. Referring to the S_BET_ value, the order of the porosity of all the samples was as follows: Zn−AC > Fe−AC > AC > Cu−AC.

#### 3.2.2. FTIR

**Figure 4:**
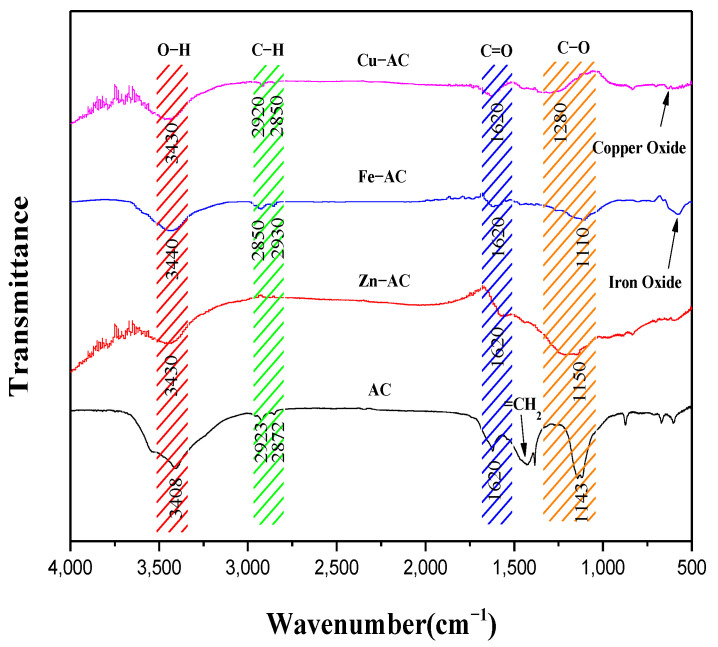
The FTIR spectra of the samples.

Figure 4 illustrates the FTIR spectra of all the samples. As shown in Figure 4, the band near 3430 cm^−1^ could be associated with the −OH bond [33], the band at around 2850 cm^−1^~2930 cm^−1^ could be associated with the aliphatic C−H bond [34], and the peaks of Zn−AC at this band were weak, indicating that its surface had higher degrees of carbonization. The band near 1428cm^−1^ in AC may be = CH_2_ of aromatic compounds [35]. The band near 1620 cm^−1^ could be associated with the C=O bond [36], and the band at around 1100 cm^−1^~1350 cm^−1^ could be associated with the C−O or C−C bond [37]. The band at about 600 cm^−1^ ~1000 cm^−1^ could be associated with an aromatic C−H bond [38]. The peaks near 500 cm^−1^~600 cm^−1^ for Fe−AC and Cu−AC could be considered to be associated with iron oxide and copper oxide, which was consistent with the SEM images and TG−DTG analysis results.

#### 3.2.3. XPS

**Figure 5:**
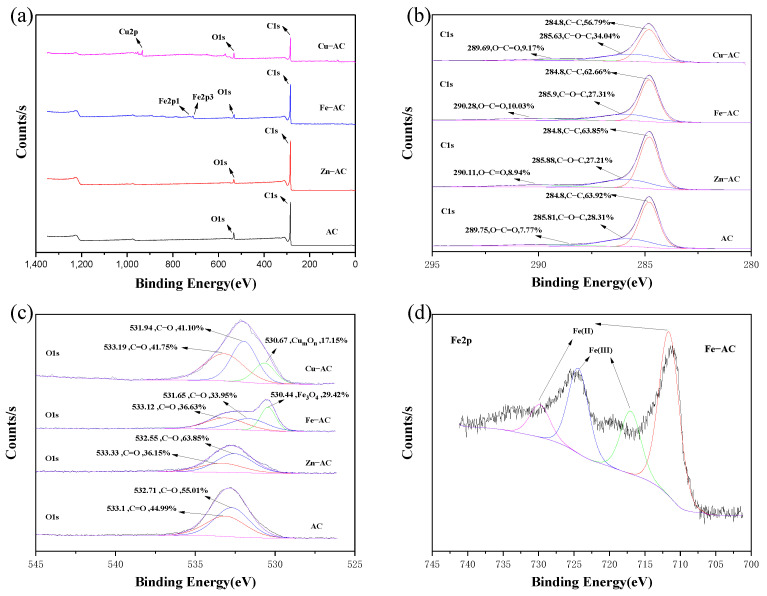
The XPS spectra of the samples: (**a**) XPS total survey spectra; (**b**) C1s; (**c**) O1s; (**d**) Fe2p in Fe−AC.

To further explore the differences between the functional groups of the different samples, XPS spectra analysis was used on the samples treated with different activating reagents. From the total survey spectra (Figure 5a), it was derived that the main elements of AC were C and O, with atomic percentage values of 92.81% and 7.19%; the main elements of Fe−AC were C, O, and Fe, with atomic percentage values of 93%, 5.25%, and 1.75%; the main elements of Cu−AC were C, O, and Cu, with atomic percentage values of 84.85%, 11.21%, and 3.94%. These illustrated that Fe^3+^ and Cu^2+^ were involved in the pyrolysis process of WPT, and Fe and Cu were attached to the surface of Fe−AC and Cu−AC, confirming the results of SEM images and FTIR spectra. The main elements in Zn−AC were C and O, with atomic percentages values of 95.92% and 3.94%, and the content of Zn was extremely low (with atomic percentages values of 0.14%), which further confirmed that ZnCl_2_ was almost completely evaporated or dissolved after pyrolysis and acid treatment.

According to Figure 5b, the C1s of AC, Zn−AC, Fe−AC, and Cu−AC could be divided into three peaks attributed to the C−C bond, the C−O bond, and the C=O bond, and the carbon−containing functional groups of the four samples had no obvious change. As shown in Figure 5c, the O1s of AC and Zn−AC could be divided into the two peaks of the C−O bond and C=O bond, while Fe−AC and Cu−AC had two more peaks of Fe_3_O_4_ and Cu_m_O_n_. The proportion of the oxygen−containing functional groups of the four samples was quite different, which caused the difference in the adsorption capacity of the four samples for Cr(VI). According to Figure 5d, the Fe2p of Fe−AC were divided into two doublet peaks, the Fe(III) species at 717.0 eV and 724.4 eV, and the Fe(II) species at 711.6 eV and 729.8 eV, which indicated that there were two valence states of Fe on the AC surface, confirming the generation of Fe_3_O_4_ on the surface of Fe−AC.

Based on the FTIR and XPS analyses, abundant acidic surface functional groups were formed during the pyrolysis process of WPTs, which played a key role in the adsorption of Cr(VI). After the addition of active reagents, Fe_3_O_4_ and Cu_m_O_n_ were generated on the Fe−AC surface and Cu−AC surface, which also promoted the adsorption of Cr(VI) by increasing the number of adsorption sites.

### 3.3. Adsorption of Cr(VI)

#### 3.3.1. Effects of pH

**Figure 6:**
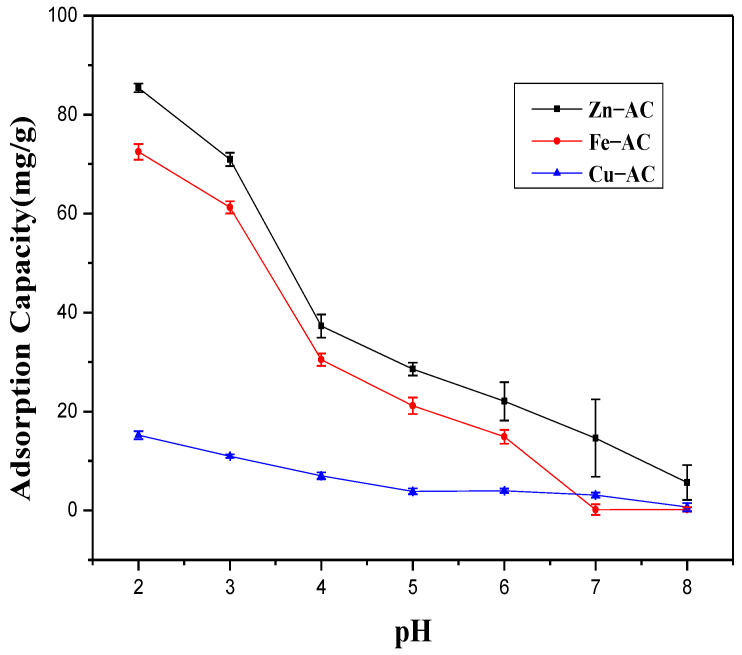
The effect of pH on the adsorption capacities of Cr(Ⅵ) onto the samples.

**Figure 7:**
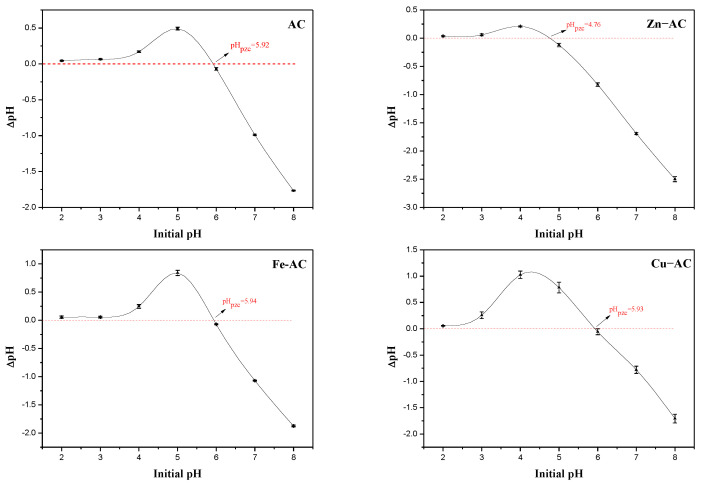
The pH_PZC_ determination curves of the samples.

One of the key factors affecting the effectiveness of Cr(VI) removal was the pH value of the solution. As depicted in Figure 6, the maximum adsorption capacities for Cr(VI) of Zn−AC, Fe−AC, and Cu−AC at pH = 2.0 were 85.395, 72.495, and 15.216 mg/g, respectively. However, the maximum adsorption capacities for the total Cr of Zn−AC, Fe−AC, and Cu−AC at pH = 2.0 were 87.508, 77.783, and 23.617 mg/g. The differences indicated that part of Cr(VI) was reduced to Cr (III) during the adsorption process. The adsorption capacities of Zn−AC, Cu−AC, and Fe−AC showed a decreasing trend with the increase in the initial pH value. The experimental pHpzc values of Zn−AC, Fe−AC, Cu−AC, and AC were observed as 4.76, 5.94, 5.93, and 5.92, respectively (Figure 7). This indicated that the surface of the ACs exhibited electropositivity, which may be due to the protonation of the surface when the pH value of solutions was around 2–3 [39]. By contrast, the main forms of Cr(VI) in the solutions were the negatively charged chromate ion (CrO_4_^2−^) and the hydrochlorate ion (HCrO_4_^−^). Therefore, the ions with negative electrons would be adsorbed by the ACs due to electrostatic interactions, and all the materials had the best adsorption performance in this pH range. The surface of the ACs carried a negative charge with the pH value increasing, and the adsorption performance became worse due to electrostatic repulsion [16]. This trend proved the existence of an electrostatic effect for the adsorption of Cr(VI).

#### 3.3.2. Adsorption Isotherms

**Figure 8:**
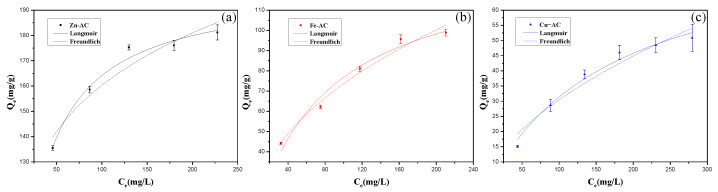
The adsorption isotherm curves of Cr(Ⅵ) onto the samples: (**a**) Zn−AC; (**b**) Fe−AC; (**c**) Cu−AC.

The data for the adsorption of Cr(VI) on the ACs were fitted using the Langmuir and Freundlich models, and the adsorption isotherm fitting results are shown in Figure 8, and the relevant parameters of the two models are summarized in Table 2. According to the R^2^ values fitted by using the two models, the adsorption behavior of Zn−AC and Cu−AC for Cr(VI) conformed to the Langmuir model (with R^2^ both of 0.98), which indicated that the adsorption behavior of Zn−AC and Cu−AC for Cr(VI) was mainly affected by monolayer coverage [40,41]. By contrast, the adsorption behavior of Fe−AC for Cr(VI) mainly corresponded to a multilayer adsorption manner, because the fitting results were more inclined to the Freundlich model (with R^2^ of 0.97) [2]. The adsorption capacities of Cr(VI) were 199.07, 136.25, and 84.47 mg/g, respectively. The adsorption intensity R_L_ was calculated to evaluate the adsorption favorability [42], and the calculation equation is as follows:

(15) $$R_{L}=1\;/\;\left(1+{\;C}_{0}K_{L}\right)\;$$

where C_0_ is the initial Cr(VI) concentration of the solution. The adsorption behavior is irreversible when R_L_ = 0, favorable when 0 < R_L_ < 1, linear when R_L_ = 1, and unfavorable when R_L_ > 1 [43].

According to the R_L_ values of the three samples (with the value of 0.299, 0.606, and 0.769), the adsorption behaviors of the ACs for Cr(VI) were all favorable [44]. Table 3 provides the maximum adsorption capacities of the activated carbons derived from different materials for Cr(VI) in some of the studies in the literature.

The maximum adsorption capacities of Zn−AC, Fe−AC, and Cu−AC for Cr(VI) in this study were higher than many other ACs [45,46,47,48,49]. This showed that the catalytic pyrolysis of the metal cations carried by the WPT itself to prepare activated carbon for the removal of hexavalent chromium from polluted water has a promising development prospect.

#### 3.3.3. Adsorption Kinetics

**Figure 9:**
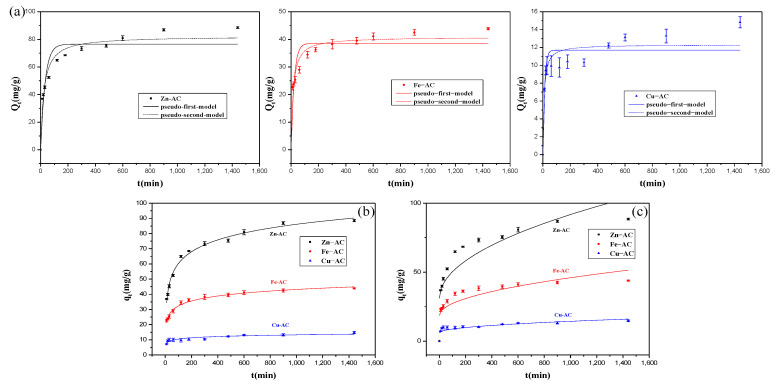
The adsorption kinetic curves of Cr(Ⅵ) onto the samples: (**a**) PFO and PSO; (**b**) Elovich; (**c**) intra−particle diffusion.

The fitting results of PFO, PSO, Elovich, and intra−particle diffusion kinetic models for the adsorption behavior of the ACs for Cr(VI) at different initial concentrations are shown in Figure 9. Table 4 summarizes the relevant kinetic parameters. The results showed that the PSO model had a higher R^2^ value than the PFO model, and the q_e_ calculated by this model was more closed to the corresponding q_e, exp_, which indicated that the adsorption behavior of Zn−AC, Fe−AC, and Cu−AC for Cr(VI) was more suiting with the PSO kinetic model, and this adsorption behavior may be related to the chemical adsorption mechanism [52]. The Elovich model had the highest R^2^ value for Zn−AC and Fe−AC, which also illustrated that the adsorption mechanism of Zn−AC and Fe−AC was chemical adsorption. According to the adsorption rate constant (k_2_), Cu−AC could remove Cr(VI) in the shortest time, while Fe−AC and Zn−AC were relatively slow [28].

### 3.4. Adsorption Mechanisms

Based on the above analysis, the maximum adsorption capacities of Zn−AC, Fe−AC, and Cu−AC for Cr (VI) were 199.07, 136.25, and 84.47 mg/g, respectively. High porosity and the existence of metal oxides on the surface of the ACs provided many adsorption sites for Cr(VI). The existence of an electrostatic interaction was confirmed by analyzing the effect of pH on the adsorption behavior. The FTIR results indicated that abundant surface functional groups existed on the surface of the ACs, which played a key role in the removal of Cr(VI).

**Figure 10:**
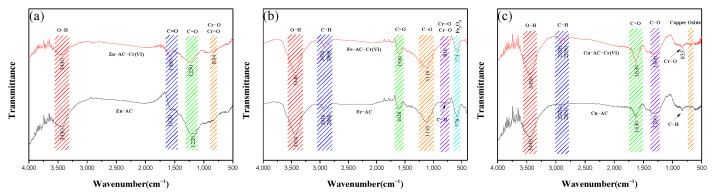
The FTIR spectra of the samples before and after adsorption of Cr(Ⅵ): (**a**) Zn−AC; (**b**) Fe−AC; (**c**) Cu−AC.

As shown in Figure 10, the FTIR spectra of Zn−AC, Fe−AC, and Cu−AC after Cr(VI) adsorption were measured to study the role of surface functional groups during the adsorption process. For Zn−AC, the peaks at 3430 cm^−1^, 1620 cm^−1^, and 1220 cm^−1^ shifted to 3440 cm^−1^, 1580 cm^−1^, and 1250cm^−1^, respectively, which corresponded to the changes in the −OH bond, the C=O bond, and the C−O bond, implying that Cr(VI) interacted with these functional groups during the adsorption process. For Fe−AC, there was no obvious change at other peaks except for the peaks at 3430 cm^−1^ and 1620 cm^−1^, which indicated that Cr(VI) mainly interacted with the −OH and C=O bonds during the adsorption process. For Cu−AC, the −OH and C−O bonds mainly participated in the adsorption process, because the peaks at 3440 cm^−1^ and 1290 cm^−1^ noticeably changed. In the FTIR spectra of the ACs after Cr(VI) adsorption, a peak appeared near 800–835 cm^−1^, which corresponded to the Cr−O or Cr=O bond [53]. This indicated that Cr(VI) was successfully adsorbed onto the surface of the ACs.

As shown in Figure S1, the ACs after Cr(VI) adsorption were analyzed with XPS. According to Figure S1a, Cr2p appeared in the survey spectra of the adsorbed Zn−AC, Fe−AC, and Cu−AC, which also confirmed that Cr(VI) was successfully adsorbed onto the surface of the ACs. Figure S1b shows the high−resolution Cr2p narrow−spectrum scanning of Zn−AC, Fe−AC, and Cu−AC after Cr(VI) adsorption. The characteristic peaks of Cr(III) appeared near 578 eV and 587 eV, and those of Cr(VI) appeared near 580 eV and 589 eV, which indicated the existence of a reduction reaction during the Cr(VI) adsorption process [9]. Under the condition of a low pH value, the oxygen−containing functional group would be protonated, which caused part of Cr(VI) to convert to Cr(III) [54]. The reduction reactions of Cr(VI) are as follows [9,54]:

(16) $${\mathrm{HCrO}}_{4}^{-}+7H^{+}+3e^{-}\to {\mathrm{Cr}}^{3+}+4H_{2}O$$

(17) $${\mathrm{CrO}}_{4}^{2-}+8H^{+}+3e^{-}\to {\mathrm{Cr}}^{3+}+4H_{2}O$$

**Figure 11:**
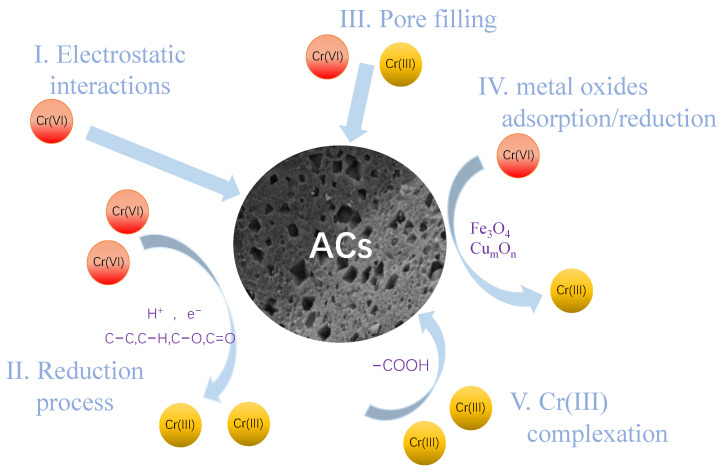
The adsorption mechanism of Cr(Ⅵ) onto Zn−AC, Fe−AC, and Cu−AC.

Zn−AC exhibited the highest reducing power of 85.13% for Cr(VI). Meanwhile, 83.21% and 54.72% of Cr(VI) on the surfaces of Fe−AC and Cu−AC were reduced to Cr(III), respectively. This could be explained by the metal standard reduction potential. The values for the standard reduction potential of Fe^3+^/Fe^2+^ (+0.77 V), Fe^2+^/Fe^0^ (−0.44 V), Cu^2+^/Cu^0^ (+0.34 V), and Zn^2+^/Zn^0^ (−0.76 V) were less positive than that of Cr^6+^/Cr^3+^ (+1.51 V), which indicated that Fe^0^, Fe^2+^, Cu^0^, and Zn^0^ can reduce Cr(VI) to form Cr(III) [55]. As shown in Figure S1c, the peaks near 284.8 eV, 286.0 eV, and 290.0 eV could be associated with the C−C bond, the C−O bond, and the C=O bond [54]. After adsorption, the relative proportion of the C−C bond of the ACs slightly decreased, and that of the C−O bond slightly increased. This was owing to the C−C bond being oxidized by Cr(VI) and forming the C−H bond, followed by the C−H bond being further oxidized to the C−O bond [56,57]. As shown in Figure S1d, the intensity of the peaks corresponding to the monodentate and bidentate carboxylate oxygen atoms at around 532 eV and 533 eV [29] after the adsorption of the ACs was changed, probably due to the newly formed Cr^3+^ combined with the negatively charged carboxylate groups via electrostatic attraction or complexation [58]. The intensity of the peaks corresponding to Fe_3_O_4_ in Fe−AC and Cu_m_O_n_ in Cu−AC decreased significantly. This indicated that the metal oxides were oxidized and gradually converted to higher species, which confirmed that Fe_3_O_4_ and Cu_m_O_n_ participated in the adsorption behavior of Fe−AC and Cu−AC for Cr(VI). Furthermore, a new peak appeared near 536 eV in the ACs, which corresponded to Cr−O [59]. This is also evidence that Cr(VI) was adsorbed on the ACs. The adsorption mechanism of the ACs for Cr(VI) is shown in Figure 11.

## 4. Conclusions

In this study, we researched the effects of the catalysis of the Zn^2+^, Fe^3+,^ and Cu^2+^ on the pyrolysis of WPTs. The pyrolysis properties of raw materials with different metal ions, surface morphologies, pore structures, and surface functional groups of their pyrolysate were characterized. The adsorption behavior of the ACs (Zn−AC, Fe−AC, and Cu−AC) for Cr(VI) was confirmed. (1) The addition of the three metal ions decreased the decomposition temperature of WPTs, where Zn^2+^ had the most significant effect. (2) The ACs possessed well−developed pore structures: Cu−AC mainly possessed a microporous structure, Fe−AC mainly possessed a mesoporous structure, Zn−AC possessed a micro−mesoporous structure, and Zn−AC had the greatest surface area, reaching 847.87 m^2^/g. (3) The generated functional groups on the surface of the ACs were involved in the adsorption process, and the Fe_3_O_4_ and Cu_m_O_n_ generated on the Fe−AC and Cu−AC surfaces were favorable for Cr(VI) removal. (4) The adsorption of Cr(VI) was more favorable at low pH conditions, and the maximum adsorption capacities of Zn−AC, Fe−AC, and Cu−AC for Cr(VI) were 199.07, 136.25, and 84.47 mg/g. (5) The adsorption mechanism includes pore filling, electrostatic effect, reduction reaction, and complexation. The study on the pyrolysis and adsorption behavior of the activated carbon derived from different kinds of waste textiles will be conducted in the future.

## Figures and Tables

**Table 1:** Textural properties of activated carbon derived from WPTs.

Sample	S_BET_ (m^2^g^−1^)	S_mic_ (m^2^g^−1^)	S_ext_ (m^2^g^−1^)	S_mic_/S_BET_ (%)	S_ext_/S_BET_ (%)	V_t_ (cm^3^g^−1^)	V_mic_ (cm^3^g^−1^)	V_mic_/V_t_ (%)	D_p_ (nm)
AC	548.89	516.10	32.79	94.03	5.97	0.213	0.197	92.49	1.55
Zn−AC	847.87	680.02	167.85	80.20	19.80	0.452	0.274	60.62	2.13
Fe−AC	229.66	101.42	128.24	44.16	55.84	0.452	0.043	9.51	7.87
Cu−AC	98.33	94.65	3.68	96.26	3.74	0.043	0.039	90.70	1.74

**Table 2:** Parameters of the isotherm models for Cr(VI) adsorption onto activated carbon derived from WPTs.

Adsorbent	Langmuir				Freundlich		
	q_m_ (mg/g)	K_L_ (L/mg)	R_L_	R^2^	K_F_ (mg/g)	n	R^2^
Zn−AC	199.07	0.047	0.299	0.98	71.63	5.72	0.91
Fe−AC	136.25	0.013	0.606	0.96	9.45	2.24	0.97
Cu−AC	84.47	0.006	0.769	0.98	2.32	1.79	0.94

**Table 3:** Comparison of the maximum adsorption capacities of different adsorbents for Cr(VI).

Adsorbents	q_m_(mg/g)	References
Peanut−hull−activated carbon	77.5	[45]
Bamboo−bark−activated carbon	19.53	[46]
Magnetic bamboo−activated carbon	75.8	[47]
Activated carbon from corncob	208.6	[48]
Melia azedarach wood−activated carbon	25.27	[49]
Raspberry−stalk−activated carbon	16.3	[50]
Activated carbon from groundnut husk	131	[51]
Zn−AC	199.07	This study
Fe−AC	136.25	This study
Cu−AC	84.47	This study

**Table 4:** Parameters of the kinetic models for Cr(VI) adsorption onto activated carbon derived from WPTs.

Kinetic Model	Sample	Parameters			
Pseudo−first−order		q_e,exp_ (mg·g^−1^)	q_e_ (mg·g^−1^)	k_1_ (min^−1^)	R^2^
	Zn−AC	88.54	76.51	0.031	0.87
	Fe−AC	43.88	38.56	0.047	0.86
	Cu−AC	14.83	11.70	0.084	0.82
Pseudo−second−order		q_e,exp_ (mg·g^−1^)	q_e_ (mg·g^−1^)	k_2_ (g·mg^−1^·min^−1^)	R^2^
	Zn−AC	88.54	82.30	0.0005	0.95
	Fe−AC	43.88	40.86	0.0017	0.95
	Cu−AC	14.83	12.33	0.0102	0.87
Elovich		α (mg·g^−1^·min^−1^)		β (g·mg^−1^)	R^2^
	Zn−AC	23.99		0.089	0.99
	Fe−AC	46.11		0.213	0.98
	Cu−AC	68.26		0.825	0.83
Intra−particle diffusion		k_3_ (g·mg^−1^·min^−1/2^)		C	R^2^
	Zn−AC	1.919		31.12	0.75
	Fe−AC	0.865		18.64	0.65
	Cu−AC	0.258		6.24	0.62

## Data Availability

The datasets used and/or analyzed during the current study are available from the corresponding author upon reasonable request.

## Supplementary Materials

The following supporting information can be downloaded at: https://www.mdpi.com/article/10.3390/ma15207112/s1, Figure S1: The XPS spectra of the samples before and after adsorption of Cr(Ⅵ): (a) XPS survey spectra; (b) Cr2p; (c) C1s; (d) O1s.
