# Supplementary material for: Study on the Pyrolysis and Adsorption Behavior of Activated Carbon Derived from Waste Polyester Textiles with Different Metal Salts
Source: Materials (Basel). 2022 Oct 13;15(20):7112. doi: 10.3390/ma15207112 (PMC9605055; doi:10.3390/ma15207112)
Supplement: Supplementary file 1 [file materials-15-07112-s001.zip › materials-1945064-supplementary.pdf]

Study on the pyrolysis and adsorption behavior of activated carbon derived from waste polyester textiles with different metal salts

Lun Zhou [1], Meng-Qi Zhong [1], Teng Wang [1,2], Jing-Xin Liu [1,2], Meng Mei [1,2], Si Chen [1,2,*] and Jin-Ping Li [1,2,*]

1 School of Environmental Engineering, Wuhan Textile University, Wuhan 430073, China
2 Engineering Research Centre for Clean Production of Textile Dyeing and Printing, Ministry of Education, Wuhan Textile University, Wuhan 430073, China
* Correspondence: wtu2019098@163.com (S.C.); lijinping@wtu.edu.cn (J.-P.L.)

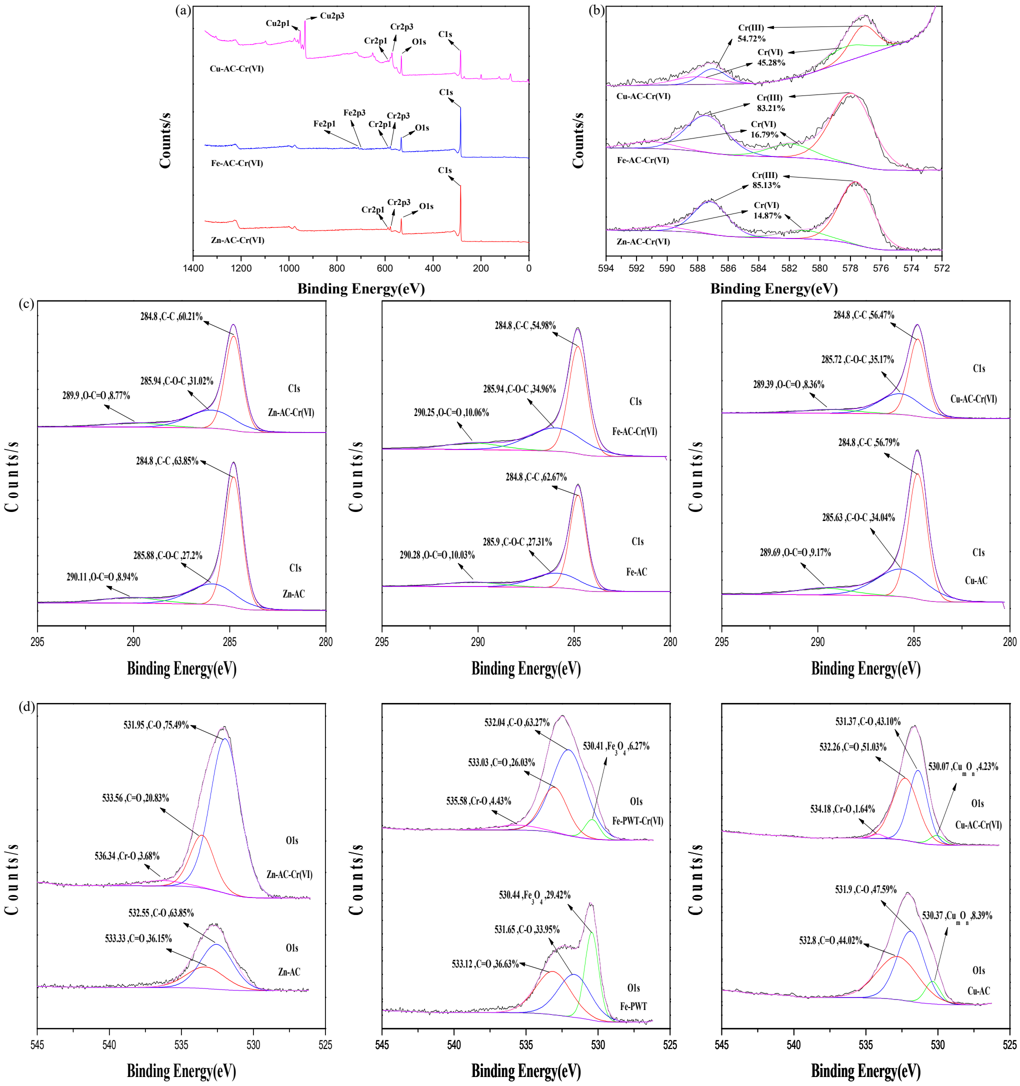

Figure S1. The XPS spectra of the samples before and after adsorption of Cr(VI): (a) XPS survey spectra; (b) Cr2p; (c) C1s; (d) O1s
